# Enhancing Microcalcification Detection in Mammography with YOLO-v8 Performance and Clinical Implications

**DOI:** 10.3390/diagnostics14242875

**Published:** 2024-12-20

**Authors:** Wei-Chung Shia, Tien-Hsiung Ku

**Affiliations:** 1 Molecular Medicine Laboratory, Department of Research, Changhua Christian Hospital, Changhua 500, Taiwan; 2 School of Big Data and Artificial Intelligence, Fujian Polytechnic Normal University, Fuqing 350300, China; 3 Artificial Intelligence Development Center, Changhua Christian Hospital, Changhua 50050, Taiwan; 21203@cch.org.tw; 4 Department of Applied Chemistry, Providence University, Taichung 43301, Taiwan

**Keywords:** microcalcification, mammography, deep learning, object detection

## Abstract

**Background**: Microcalcifications in the breast are often an early warning sign of breast cancer, and their accurate detection is crucial for the early discovery and management of the disease. In recent years, deep learning technology, particularly models based on object detection, has significantly improved the ability to detect microcalcifications. This study aims to use the advanced YOLO-v8 object detection algorithm to identify breast microcalcifications and explore its advantages in terms of performance and clinical application. **Methods**: This study collected mammograms from 7615 female participants, with a dataset including 10,323 breast images containing microcalcifications. We used the YOLO-v8 model for microcalcification detection and trained and validated the model using five-fold cross-validation. The model’s performance was evaluated through metrics such as accuracy, recall, F1 score, mAP50, and mAP50-95. Additionally, this study explored the potential applications of this technology in clinical practice. **Results**: The YOLO-v8 model achieved an mAP50 of 0.921, an mAP50-95 of 0.709, an F1 score of 0.82, a detection accuracy of 0.842, and a recall rate of 0.796 in breast microcalcification detection. Compared to previous similar deep learning object detection techniques like Mask R-CNN, YOLO-v8 has shown improvements in both speed and accuracy. **Conclusions**: YOLO-v8 outperforms traditional detection methods in detecting breast microcalcifications. Its multi-scale detection capability significantly enhances both speed and accuracy, making it more clinically practical for large-scale screenings. Future research should further explore the model’s potential in benign and malignant classification to promote its application in clinical settings, assisting radiologists in diagnosing breast cancer more efficiently.

## 1. Introduction

Breast microcalcifications are a common finding on mammography, usually referring to the presence of calcium deposits smaller than 0.5 mm in diameter within breast tissue. The mechanism of calcium deposition is not yet clear; it is generally speculated to be caused by an active cell differentiation process. The main components are calcium oxalate and calcium phosphate [1,2]. Breast calcifications are often associated with abnormal proliferation of breast tissue. These calcifications may result from abnormal metabolism in breast cells, reflecting irregularities in cell growth and division, and thus could be early signs of precancerous lesions. Pathological examinations reveal that these calcifications display irregular shapes and arrangements under the microscope, suggesting potential malignant transformations. However, most breast calcifications are benign.

Approximately 90% of non-palpable ductal carcinoma in situ (DCIS) and 20% of microcarcinomas (invasive carcinomas <0.5 cm and all DCIS) are diagnosed solely on the basis of microcalcifications [3,4]. Microcalcifications in the breast are typically discovered during mammography and often appear as white dots or small specks. If microcalcifications are found on a mammogram, doctors usually recommend further examination such as a breast ultrasound or a needle biopsy to determine whether they are cancerous. Since microcalcifications are a common sign of breast cancer, their detection is very important in the clinical practice for breast cancer management.

Currently, breast microcalcifications on mammography are detected using two primary methods: (1) Manual detection: This is the traditional method of detecting breast microcalcifications. Radiologists examine images from mammography and determine whether they are benign or malignant based on characteristics such as the size, shape, and distribution of the microcalcifications. (2) Automatic detection: This involves using computer-aided detection systems to identify breast microcalcifications or determining their nature—benign or malignant—based on morphological features [5,6]. The advantage of manual detection is its high accuracy, but it requires a large number of radiologists to complete the task. The advantage of automatic detection is its efficiency; however, its accuracy may not be as high as that of manual detection. Currently, the detection of breast microcalcifications primarily employs a combination of both manual and automatic methods. Manual detection is used for complex cases of microcalcification while automatic detection helps in identifying simpler forms morphologically. Therefore, an efficient automated system for detecting breast microcalcifications can enhance overall screening efficiency, reduce the workload of radiologists, and make the entire diagnostic process smarter while minimizing errors associated with manual examinations.

Research on computer-aided diagnosis and detection of breast microcalcifications has been underway for some time now, with the main methods used falling into several categories: (1) Feature extraction methods: extracting features from images of breast microcalcifications, including shape, size, edge, gray level, etc. [7,8] (2) Pattern matching methods: these methods use known patterns of breast microcalcifications to detect them [9,10]. Common patterns include circular, linear, polygonal, and others. Traditional computer graphics methods were not designed to identify features in large datasets with diverse characteristics; these methods lack generalization capabilities. Since feature extraction relies on local features from the source image to find similar ones in the target image, it struggles with detecting multiple objects and inefficiencies in managing different item categories. Pattern matching requires precise templates or reference images to locate objects in the target image. When detected features undergo deformation or rotation, or when feature areas are partially occluded or incomplete, it can lead to a decline in detection performance. These issues are not uncommon in mammography images. Moreover, with the increasing number of breast cancer cases and the trend towards younger patients, there is an urgent need for a new technology and method that can process large volumes of image data and even achieve real-time detection, while offering advantages in both speed and accuracy.

Although automated detection methods have advanced, existing approaches such as Mask R-CNN still face challenges in balancing detection accuracy, computational efficiency, and the ability to identify extremely small targets like microcalcifications. These limitations underscore the need for a more robust model capable of real-time performance and accurate small-object detection. Recent advancements in object detection algorithms [11], particularly deep learning models [12], have revolutionized medical imaging analysis by enabling accurate detection of complex features such as microcalcifications. Models like Mask R-CNN and Faster R-CNN have been widely adopted [13]; however, their reliance on region proposals and multi-stage architectures often limits their speed and scalability for large-scale clinical applications.

YOLO-v8, the latest advancement in the YOLO (You Only Look Once) series, marks a significant improvement in addressing these challenges. Its single-stage design, multi-scale detection capabilities, and computational efficiency make it particularly well-suited for detecting small targets in high-resolution medical images. These characteristics align with the requirements for microcalcification detection in mammography, where accurate localization of minute features is critical for early cancer diagnosis. This study addresses this gap by employing YOLO-v8, a state-of-the-art single-stage object detection model. By optimizing for speed and multi-scale detection capability, this research advances the field by bridging the gap between experimental techniques and practical clinical applications.

## 2. Materials and Methods

**Figure 1:**
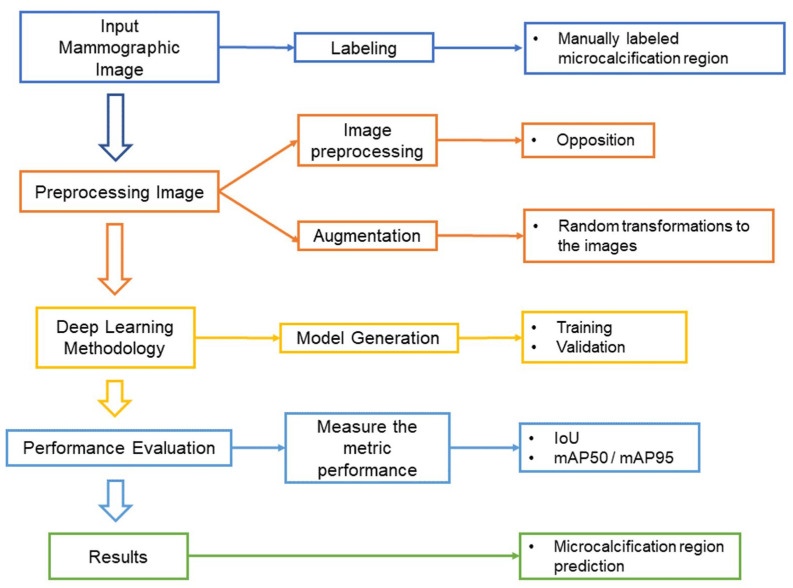
Flowchart of this study.

This study was approved by the Institutional Review Board (IRB) of Changhua Christian Hospital, Changhua, Taiwan (No. 220204). Informed consent requirement was waived by the ethics committee because of the retrospective nature of the study. The overall research process of this study is shown in Figure 1. It includes routine data collection, image preprocessing, model training, performance evaluation, etc. Relevant details will be described in detail in the following paragraphs.

### 2.1. Study Cohort

This study is retrospective research. The research methods used have been approved and supervised by the ethics review committee of the institution to which the research belongs and were conducted in accordance with relevant guidelines and the Declaration of Helsinki. Participants were selected from the breast cancer registry and mammography screening data maintained by our institution between January 2015 and December 2020. The inclusion criteria were as follows: (1) women aged 35–70 years who underwent mammography at our hospital; (2) availability of mammogram records and corresponding imaging reports that clearly indicate the presence or absence of breast microcalcifications; and (3) each participant must include images of at least one breast from both the cranio-caudal (CC) view and the mediolateral oblique (MLO) view. Exclusion criteria included: (1) mammographic images that contain artifacts such as prostheses or metal markers that affect the judgment of microcalcifications; (2) mssing mammography reports; and (3) patients who underwent breast biopsy due to microcalcifications but lack corresponding pathological tissue reports.

### 2.2. Data Acquisition

All breast imaging images were obtained from several full-field digital mammography (FFDM) systems, including a Senographe Essential (GE Medical Systems, Chicago, IL, USA) (accounts for 54.9% of all image sources) and Selenia Dimensions (HOLOGIC, Inc., Marlborough, MA, USA) (accounts for 45.1% of all image sources). The acquisition of the images was assisted by experienced physicians or assistants according to the uniform operating procedures issued by the institution to ensure consistency in the process. All acquired images were obtained in their original resolution (1914 × 2294 pixels) and color depth (16 bit), in grayscale digital imaging and communications in medicine (DCM) format. Corresponding imaging reports and medical records were also obtained along with the images.

### 2.3. Image Pre-Processing and ROI Definition

In the aspect of image preprocessing, image opposition transformation [14] was used to enhance the images, highlighting irregular areas and making regions of microcalcifications in the breast easier to identify. Due to the diversity in microcalcification image characteristics, two experts with clinical experience identified and circled areas of calcifications. After reviewing and discussing these circled results, a consensus was reached on determining the range for microcalcification selection. In training images, ROI regions are designated as areas containing clusters of microcalcifications.

### 2.4. Object-Detection Model

Currently, object detection technologies based on deep learning or deep neural network techniques have several well-known and open-source models available for evaluation. Among them, YOLO is one of these algorithms that has been widely applied due to its renowned performance in object detection [15,16,17]. It is known for its speed and efficiency, making it suitable for real-time object detection tasks. YOLO represents a series of object detection algorithms used in computer vision; the core idea is to accomplish object detection in one go through a neural network. Compared to traditional methods that require multiple processing steps, this approach significantly increases speed. By using CNNs, it directly predicts bounding boxes and their class probabilities from the entire image at once. This method differs from previous approaches that required generating region proposals in stages before classification.

In this study, we used the object detection model YOLO-v8, which is the latest version among all YOLO-related algorithms [18]. Compared to earlier YOLO algorithms, the main improvements and enhancements of YOLO-v8 include the following: (1) YOLOv8 achieves improved speed and accuracy through innovations like its Anchor-free mechanism, decoupled head, and optimized Path Aggregation Network (PANet). (2) It enhances its multi-scale detection capability, providing more accurate predictions of object size and location. (3) It uses an updated combination of architectures that can more effectively capture image features and perform classification. These improvements to architectural combinations include deeper network layers, more efficient feature extraction methods, and more precise anchor box prediction mechanisms. Compared to object detection technologies such as Mask R-CNN [19] or Fast R-CNN [20], the YOLO series of algorithms have some characteristics suitable for detecting microcalcification clusters in mammography: (1) The size of breast calcification points relative to the entire breast area is just a very small region. Even if the entire microcalcification cluster area is taken into account, it still belongs to a smaller target compared to the whole image range. The YOLO series algorithm has multi-scale detection capabilities and can detect large and small objects at the same time. And this capability is lacking in Fast R-CNN or Mask R-CNN. (2) YOLOv8, with its single-stage architecture, eliminates the need for region proposal steps in models like Mask R-CNN or Fast R-CNN, resulting in faster inference and improved small-object detection capabilities. (3) In some cases, microcalcification clusters may appear scattered across multiple locations on the breast rather than having only one or two targets. YOLO predicts objects directly on the entire image without needing to generate regional proposals, making it more suitable for full-image detection.

**Figure 2:**
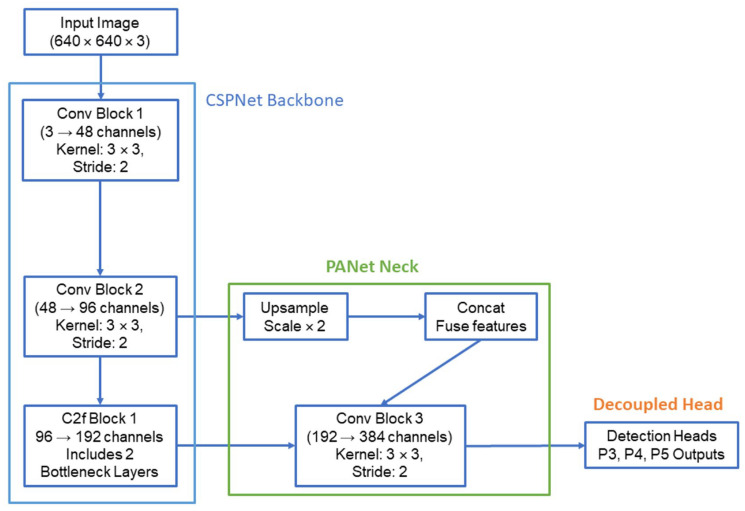
YOLO-v8 network architecture.

The backbone of YOLOv8 is a custom CSPNet-based architecture, optimized for lightweight computation and efficient multi-scale feature extraction, incorporating advancements that cater specifically to the Anchor-free detection mechanism and improve over the heavier CSPDarkNet design in YOLOv4. YOLOv8 employs an Anchor-free mechanism, removing the dependency on pre-defined anchor boxes, which improves detection precision for small objects and simplifies model design. YOLOv8 achieves multi-scale detection through its PANet, which fuses feature maps from different layers, enhancing its ability to detect small and large objects simultaneously. This is particularly beneficial for detecting microcalcifications, which occupy a very small fraction of the mammogram image. This architecture adheres to convolutional neural network (CNN) principles fundamental to modern image processing and object detection models. YOLOv8 employs a comprehensive set of data augmentation techniques, including mosaic, Copy-Paste, random scaling, rotation, and flipping, to enhance robustness and generalization. Techniques like mosaic and Copy-Paste are particularly effective for improving detection of small objects such as microcalcifications. The architecture is highly modular and supports a decoupled head design, which separates object classification from bounding box regression, collectively improving detection accuracy and speed compared to previous YOLO algorithms. Figure 2 provides a schematic of the YOLOv8 architecture, highlighting key components such as the CSP-based backbone, PANet neck, and decoupled detection head.

### 2.5. Training Protocol

Compared to previous versions of YOLO algorithms, one feature of YOLO-v8 is that it was designed with a balance between performance and accuracy in mind. During the training process, pre-trained weight models of different dimensions can be selected as the initial model according to needs. The initial model used in this study is yolov8m, which is a model specifically used for detection tasks. It can accept image input sizes of 640 × 640 pixels, has 25.9 M params, and its computational complexity is 78.9B FLOPs. When this model was pre-trained using the COCO dataset (with 80 classes) [21], its reported mean average precision (mAP) was 50.2.

During the training process, the protocol used was as follows: The initial learning rate was set to 0.01, and the stochastic gradient descent optimizer with momentum was used. Since the original image has undergone opposition transformation during the preprocessing stage, pixel normalization is no longer performed. Image augmentation techniques were applied to the dataset to enhance model adaptability; these included random scaling (from 0.8× to 1.2×), rotation (from −90° to +90°), cropping, vertical/horizontal flipping, and mosaic. Before being input into the model for training and evaluation, the images are resized to 640 × 640 pixels to meet the input requirements of the YOLO v8m model. Other training parameters included a batch size of 16 and 600 epochs total for training duration. The actual training duration was halted at 128 epochs because the accuracy did not continue to increase. The selection of these parameters is based on the default parameters provided by YOLO v8 and obtained after testing the number of images that can be accommodated by the GPU environment and its computational capacity, aiming to optimize results as much as possible and ensure a smooth training process.

In order to ensure that the model building learning process can correctly generalize image data during its establishment phase, background images (i.e., mammographic images without microcalcification annotations) need to be added in order to ensure the accuracy of the training results. According to the relevant literature of YOLO-v8, we have added about 10% blank images to improve the accuracy of the model after training and reduce false positives. The dataset was split into training, validation, and testing sets at a ratio of 7:3:1. Five-fold cross-validation was then conducted to assess the model’s robustness.

### 2.6. Performance Evaluation

The indicators used in performance evaluation included precision, recall, and mean average precision (mAP). This study uses mAP to evaluate the accuracy of detected calcification areas by models while assessing target detection performance. Here, two thresholds were utilized to establish an evaluation mean average precision at 50% (note to mAP50) and mean average precision from 50% to 95% (note to mAP50-95). Precision is the proportion of correctly detected boxes among all detected boxes, used to measure whether the model’s detections are accurate. Recall rate indicates the proportion of positive samples successfully detected by the model out of the actual targets present. The range of precision and recall is usually between 0 and 1. It is derived using TP (True Positives, the number of targets correctly detected by the model) and FN (False Negatives, the number of targets missed by the model) after calculation. mAP is used to measure the average detection precision of a model at different IoU thresholds, and it is divided into mAP50 (when the IoU threshold is fixed at 0.5) and mAP50-95 (the average value calculated stepwise from an IoU threshold of 0.5 to 0.95 in increments of 0.05). mAP50-95 is a stricter evaluation metric compared to mAP50. It provides a more comprehensive perspective of model performance since higher IoU thresholds (such as 0.75 or 0.9) require greater accuracy in bounding boxes.

### 2.7. Training Infrastructure

The computing environment used in this study is built on a virtual machine allocated by a virtual computing platform. The allocated computational resources of the virtual machine include an 8-core virtual CPU provided by Intel^®^ Xeon^®^ Gold 61 series processors (Intel, Santa Clara, CA, USA), 100 GB of virtual disk, 90 GB of virtual memory, and an NVIDIA^®^ Tesla V100 GPU (32 GB video RAM) (NVIDIA, Santa Clara, CA, USA) allocated in physical form. The operating system used is Ubuntu 20.04 LTS. The graphics processing unit acceleration computing environment is constructed using NVIDIA Compute Unified Device Architecture (CUDA) version 12.2 and NVIDIA CUDA Deep Neural Network Library version 8.9.2.26. The relevant deep learning procedures and performance evaluation metrics are implemented using PyTorch 2.0.

## 3. Results

**Figure 3:**
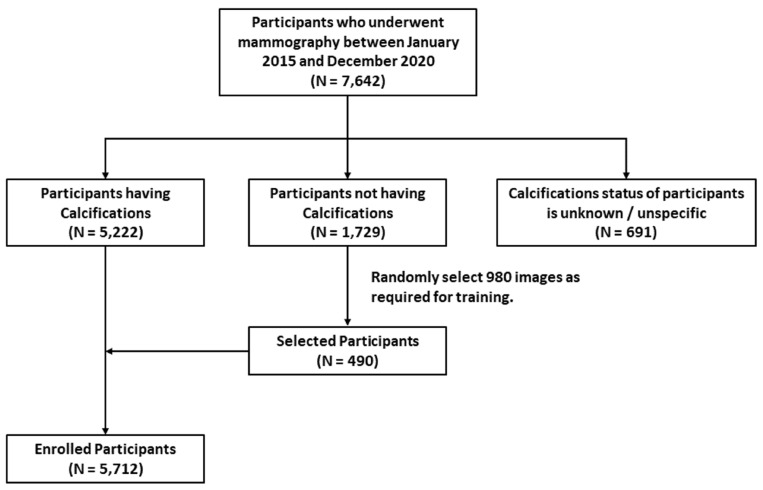
Inclusion and exclusion procedures of this study, as well as number of participants at each stage.

Between January 2016 and December 2020, data from 7642 participants who underwent mammography at the institution were collected. By analyzing imaging reports, we excluded 1729 patients whose mammography reports did not include calcifications and 691 patients whose breast calcification status was not specified or detailed in the reports. We identified a total of 5222 patients documented in the imaging reports as having breast calcifications. Among them, additional pathological biopsies and corresponding pathology reports were also obtained. After confirming the analysis of participant data, there were a total of 10,323 mammographic images containing breast microcalcifications. Regarding the blank image section mentioned earlier in the methods session, we additionally obtained 980 background images (*N* = 490) from patients who did not mention calcifications in reports and added them to the dataset. Consequently, the final image dataset comprised 5712 individuals with 11,303 breast X-ray images, where background images accounted for approximately 9.8% of the total number of images. The inclusion and exclusion processes for all participants as well as the number of participants at each stage are shown in Figure 3.

### 3.1. Characteristics of the Image Set

The basic information and quantitative statistics of the patients included in the image set are shown in Table 1. In Table 1, the breast imaging reporting and data system (BI-RADS) category and breast density information of the participants comes from records in imaging reports. The breast density assessments are based on analyses performed on original FFDM images using dedicated software (Volpara ^TM^ software version 2.0, Volpara Health Technologies, Wellington, New Zealand). The density assessment was conducted based on the volumetric density percentage (VPD) and reported according to the Volpara Density Grade (VDG), which corresponds consistently with the BI-RADS density categories. After investigating the annotated box sizes in the image set, approximately 19.1% were smaller than 48 × 48 pixels.

### 3.2. Visual Presentation of Microcalcification Detection Results

**Figure 4:**
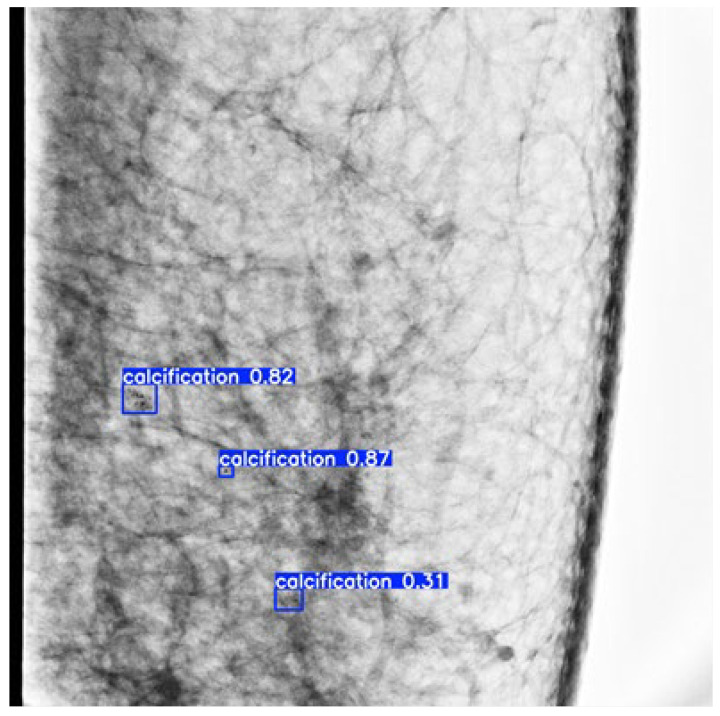
Schematic diagram of microcalcification detection results of mammography.

Several images were randomly selected from the dataset for inference tests using the trained weights. The results are shown in Figure 4. The blue box indicates the final convergence result of the generated prediction box. The blue background with white text indicates the category name (calcification) and its classification probability.

### 3.3. Performance

**Figure 5:**
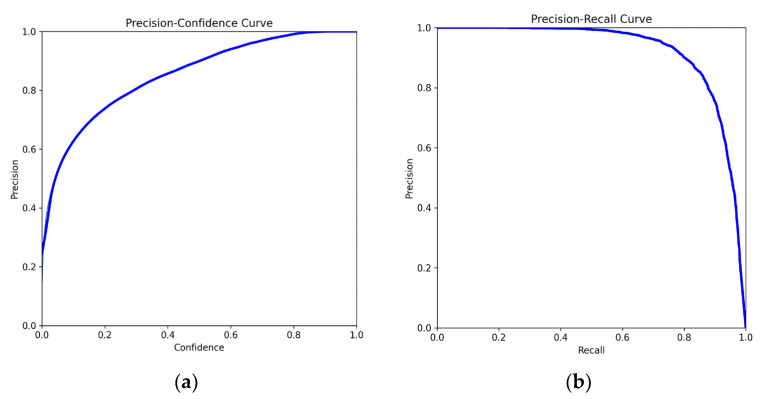
(**a**) precision–confidence curve and (**b**) precision–recall curve of model trained in this study.

**Figure 6:**
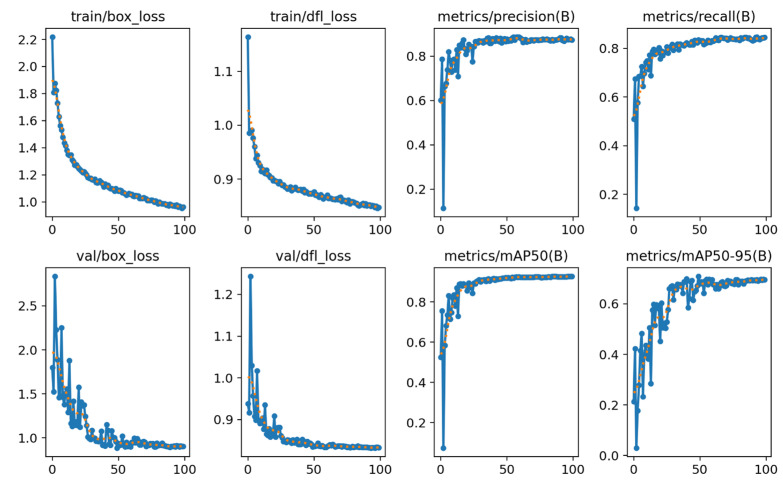
Loss curve during model training using training and validation sets. It includes bounding box loss, object presence loss, classification loss, accuracy, recall rate, and mean average precision during training periods.

The test dataset consisted of 2200 images excluded from the training set, containing 5727 regions with microcalcifications. Figure 5 shows this model’s precision/confidence curve as well as precision/recall curve, used to help observe the rate at which calcification areas are correctly predicted by the model and the model’s confidence level in its predictions. In the precision/recall curve, it can be observed that the curve is quite close to the top right corner, indicating a high prediction accuracy of the model. In terms of the analysis of the precision/confidence curve, the right end of the curve reflects high-confidence predictions by the model. When the confidence is above 0.8, the precision is close to 1, indicating reliable high-confidence predictions. In contrast, in the low-confidence area on the left side of the graph, when the confidence is below approximately 0.2, the precision is low. According to the performance evaluation of the model based on the test dataset, we obtained a precision of 0.865, recall rate of 0.836, and F1-score of 0.82. Figure 6 provides additional data on this model, such as box loss and class loss. The Train/Box_Loss curve shows a downward trend, indicating that the model’s bounding box prediction error gradually decreases with training. The Metrics/mAP50 curve rises quickly and approaches stability, suggesting that the model can accurately detect objects, especially at an IoU threshold of 0.5. The improvement in the Metrics/mAP50-95 curve indicates stable and good performance of the model across different IoU thresholds. This is a more stringent evaluation metric, and its stability reflects the overall accuracy of the model. The box loss curve and DFL loss curve in training are very close on a downward trend and both tend to stabilize, indicating that the model’s training and validation performance is consistent without obvious signs of overfitting. The mAP50 and the mAP95 of this model are 0.921 and 0.709.

**Figure 7:**
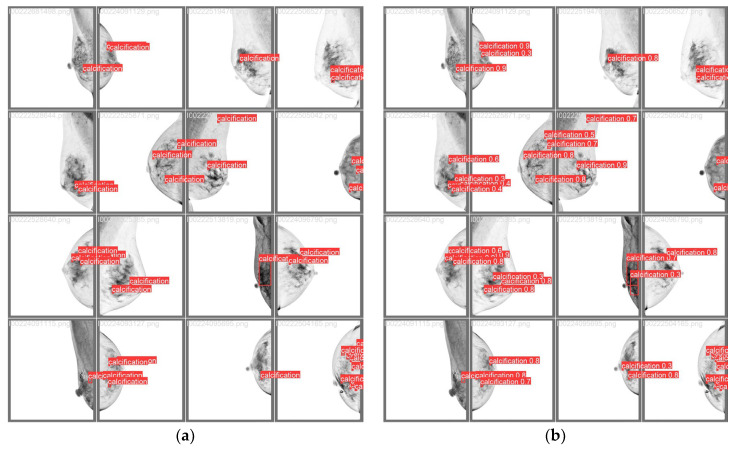
Microcalcification detection on 16 random mammography images and comparisonwith ground truth. Red labels indicate category annotations for that area. (**a**) Predicted microcalcification areas. (**b**) Ground true regions marked with microcalcifications.

Figure 7 shows a comparison between the predicted calcification point areas and actual areas from 16 randomly selected images. Figure 7a displays the real area after professional annotation, while Figure 7b presents the predicted area along with its given confidence score. It can be observed that in these random 16 images, the detected calcification point locations are almost identical, with only minor differences in some of the marked calcification point sizes.

### 3.4. Performance Comparison to Previous Algorithms

An important consideration is whether YOLO-v8 is significantly superior to previous deep learning algorithms in detecting breast microcalcifications. We tested the classic deep learning object detection network, Mask R-CNN, using the same dataset and evaluated whether there is a significant performance difference with YOLO v8 by using pre-trained weights generated from the ImageNet 10 K dataset [22]. In this experiment, we used training and testing datasets of the same size and content to evaluate Mask R-CNN’s detection of breast microcalcifications.

**Figure 8:**
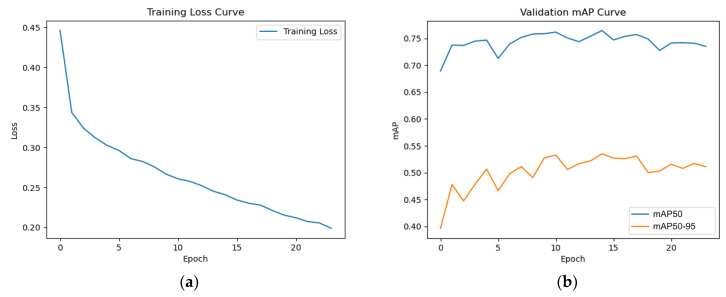
Loss curve and mAP curve during model training and validation using Mask R-CNN. (**a**) Training loss curve. (**b**) mAP curve in validation.

In terms of implementation details, we used Python 3.12 and Pytorch 2.0 for programming and referred to the original paper, using Resnet 101 with FPN as the backbone network and AdamW as the optimizer. We also set gradient clipping (max_norm = 5.0). The initial learning rate was set to 0.0001, and mixed precision training was employed to dynamically adjust the learning rate based on each epoch’s loss rate. The original image annotations in the YOLO format were converted to the training data format required for Mask R-CNN. Due to the higher video memory usage of Mask R-CNN during training compared to YOLO v8, the batch size, subdivisions, and number of iterations used have been adjusted for evaluation to accommodate the operation on cloud platforms. The test results show that Mask R-CNN achieved 76.4% mAP50 and 53.5% mAP50-90, which is significantly lower than the results obtained after training and evaluating with YOLO v8 by about 20%. Figure 8 shows the loss curve and mAP curve generated during training with Mask R-CNN. During training, we set it to run for 50 epochs, but early stopping was triggered at the 25th epoch because its mAP and loss rate no longer showed significant changes over a certain period.

## 4. Discussion

This study builds on previous research in automated microcalcification detection, addressing the limitations of two-stage detectors like Mask R-CNN in handling small calcifications and achieving real-time performance. By leveraging the YOLO-v8 model’s single-stage architecture and multi-scale detection capability, this research not only achieves higher accuracy but also significantly improves inference speed. These advancements position YOLO-v8 as a practical alternative for large-scale clinical screening tasks, filling a critical gap in the existing methodologies.

Given the importance of breast calcifications in clinical breast cancer diagnosis, detecting calcifications in breast imaging (whether based on mammography or ultrasound) has been a subject of research for several decades [23]. The earliest methods can be traced back to traditional image processing techniques based on grayscale extraction or pixel extraction, combined with clustering algorithms to segment calcification areas. Subsequently, due to advancements in signal processing, neural network technologies, and the field of machine learning, there has been a gradual inclusion of support vector machines, Bayesian networks, wavelet transformations [24,25], and other technologies [26]. Pattern matching performance typically declines as data volume grows and detection methods diversify; these traditional approaches also struggle to generalize more complex forms of microcalcifications. YOLOv8 achieves multi-scale detection by fusing feature maps from different layers through its PANet, enabling the model to detect both small and large objects more effectively. These innovations enhance its ability to identify extremely small calcifications that occupy only a fraction of the image area. Additionally, the decoupled head in YOLOv8 separates object classification from bounding box regression, minimizing task interference and enhancing both detection accuracy and inference speed.

This study tackles the issue by employing a vast image dataset and a deep-learning-based object detection method, which marks a significant departure from previous research approaches. The deep learning detection model developed in this study, when trained on a large amount of real data, significantly outperforms previous studies’ results and methods, demonstrating its practicality. Past studies used fast R-CNN [27] or mask R-CNN [28] to detect breast calcifications, with an accuracy rate of about 90%. However, most of these studies rely on publicly available image datasets for analysis, and the number of images in these public datasets typically ranges from several hundred to over a thousand [29,30]. In our experiment using Mask R-CNN as an example, when employing a real dataset and increasing the number of images to ten thousand, our evaluation showed that the mAP50 of Mask R-CNN was only about 75%. This may be because the optimization parameters of Mask R-CNN include the ability to pre-specify anchor sizes, allowing it to generate more accurate ROIs during object detection [31]. However, the shapes and sizes of breast calcifications are highly variable, making it nearly impossible to optimize by providing a consistent anchor size setting. The anchor-free mechanism in YOLOv8 simplifies the model design by eliminating the need for pre-defined anchor boxes, reducing computational overhead and improving detection precision for small objects. YOLOv8’s single-stage detection design eliminates the need for region proposals required in models like Mask R-CNN [19], significantly improving inference speed while maintaining high accuracy, especially for small object detection. YOLO-v8 demonstrates superior flexibility and adaptability, even on edge computing platforms. Based on our evaluation results on the same computing platform utilized in this study, when using a CPU-only environment for inference, each image takes about 115 microseconds to process on average; its speed is about three to four times faster than that of using Mask R-CNN (0.5 s on average).

One significant advantage of using YOLO in our research is its rapid processing speed and global reasoning capability. YOLO operates as a single-stage detector, processing images through one neural network that divides the image into multiple grids to perform bounding box predictions and classifications simultaneously across each grid. To clarify the architectural advantages of YOLO-v8, we highlight key innovations that differentiate it from Fast R-CNN and Mask R-CNN: (1) CSP-Darknet improves gradient flow and feature extraction efficiency; (2) the enhanced FPN optimizes multi-scale detection; and (3) the decoupled head improves inference speed and accuracy. Unlike Mask R-CNN, which heavily relies on region proposals, YOLO-v8’s single-stage detection design eliminates these steps, achieving faster inference and superior performance on small targets. Importantly, we note that YOLO-v8 does not employ self-attention mechanisms, focusing instead on convolutional feature extraction enhancements tailored for efficient object detection [32]. This efficiency is crucial for handling large volumes of images during both the training and inference phases. This advantage can extend to the detection and differentiation of multiple types of lesions, not just microcalcification areas. It can also aid in the real-time detection of masses or high-breast-density areas.

An often-raised question is whether the detection results from deep learning methods align completely with manual determinations. According to the evaluation results, we can observe that there is a size discrepancy between the predicted calcification areas and the actual areas. In the model’s evaluation results, mAP50-95 is 70.9% and it indicates that in all images identified as containing calcifications, about 70% show marked areas that almost overlap with the actual areas. In practice, when doctors detect microcalcifications, they also examine other suspicious features around the area. The size of the marked region does not significantly affect clinical judgment. The final diagnosis still depends on the pathological report obtained from the biopsy of the calcified tissue. At this point, the distribution pattern of the calcification fragments and cytological results become more important. Therefore, the final outcomes of such auxiliary detection technologies still require a physician’s judgment.

Due to the poor penetration of X-rays through dense breast tissue, artifacts and blurred areas are more likely in the images. A question raised is whether the model’s ability to detect calcification points in these images has declined. We randomly selected 2000 images from BI-RADS density categories C and D for performance re-evaluation, achieving an mAP50 of 0.901, indicating that high breast density does not affect the model’s discriminative ability. This is related to the image transformation already performed during preprocessing to enhance contrast.

Future research should focus on integrating classification capabilities into the detection framework to simultaneously identify and categorize microcalcifications as benign or malignant. This integration could be achieved through multi-task learning approaches, leveraging clinical data and expert annotations to enhance model performance. Furthermore, expanding the dataset to include images from diverse populations and mammography systems will improve the model’s generalization capability, ensuring its applicability across different clinical environments. By addressing these gaps, this study establishes a foundation for integrating more efficient and precise automated detection systems into clinical workflows, paving the way for future advancements in breast cancer diagnostics.

## 5. Conclusions

This study utilizes a large-scale image dataset and deep-learning-based object detection methods to overcome key challenges in automated microcalcification detection. By employing the state-of-the-art YOLO-v8 model, we significantly improve both the speed and accuracy of detection compared to traditional image pattern matching techniques and alternative deep learning models such as Mask R-CNN. Specifically, YOLO-v8 eliminates the complex candidate region generation process typical of two-stage detectors, achieving more efficient training and faster inference times. Its single-stage architecture and multi-scale detection capabilities enhance the model’s ability to identify extremely small calcifications within complex mammographic images, addressing key limitations of prior approaches.

This study contributes to the literature by demonstrating YOLO-v8’s superior performance on a large-scale, real-world dataset, effectively bridging the gap between experimental methodologies and clinical applications. Integrating this technology into existing diagnostic workflows enables radiologists to quickly pinpoint areas in mammography images requiring further examination, aiding earlier and more accurate breast cancer diagnoses. Future research will focus on expanding the dataset to include multi-center imaging data and exploring the model’s potential for classifying calcifications as benign or malignant, further enhancing its clinical utility. By addressing these gaps, this study contributes to more efficient and precise breast cancer screening, strengthening its role in improving patient outcomes.

## Figures and Tables

**Table 1:** Characteristics of participants in this study.

Characteristic	Status	Number of Participants (*N*)
With breast microcalcifications	Yes No	5222 490
BI-RADS Category	1 2 3 4 >5	286 2072 1062 1701 591
Breast Density (Grade in Volpara ^TM^ Density Grade)	A B C D	113 1259 2864 1476

## Data Availability

Image data are unavailable due to privacy and ethical restrictions. The source code for YOLO v8 can be obtained from https://github.com/ultralytics/ultralytics (accessed on 19 December 2024), along with related deployment documentation.
